# The Effect of pH on the Morphological, Thermal, and Mechanical Properties of Cross‐Linked Starch Edible Films

**DOI:** 10.1155/ijfo/5120220

**Published:** 2026-10-13

**Authors:** Yuan Chi, Melvin A. Pascall

**Affiliations:** ^1^ Department of Food Science and Technology The Ohio State University Columbus Ohio USA osu.edu

**Keywords:** citric acid, cross-linking, edible films, mechanical stability, tapioca starch

## Abstract

This study evaluated the chemical and physical properties of edible films formed by cross‐linked tapioca starch that was plasticized by glycerol. To optimize the formula of the film formation solution and enhance the mechanical and thermal properties of the material, the pH values of the solutions were adjusted to 3, 5, 7, 9, 11, respectively. The specific film properties that were evaluated included the degree of cross‐linking, crystallinity, morphology, thermal stability and mechanical functionalities. These properties were analyzed using Attenuated Total Reflection Fourier Transformation Infrared Spectroscopy (ATR‐FTIR), x‐ray diffraction (XRD), thermal gravimetric analysis (TGA), and tensile profile analysis (TPA). The ATR‐FTIR results showed that pH 7 and 9 were optimum for increasing the degree of cross‐linking. The XRD results showed that crystallinity increased with increasing pH values up to pH 11. The TGA results showed that pH 9 provided the cross‐linked starch film with optimized heat resistance. The TPA results showed that pH 9 provided the cross‐linked starch film with the highest tensile strength and elastic modulus, but with the lowest percent elongation. This research showed how the pH of a tapioca starch film forming solution could be adjusted to modify the properties of dried films subsequently produced.

## 1. Introduction

Starches are of botanical sources, and they exist as bulk polysaccharide reserves of plants. They are the second most abundant source of polysaccharides, cellulose being the first [1]. Starches are relatively low cost when compared with other sources of polysaccharides. Both native forms and modified starches are commonly used in the food industry. One such application is their use as edible films and coatings, where they are used to enhance the attributes and self life of selected foods [2].

Starch is composed of two major polysaccharides. The first is amylose, which is the linear fraction of the chain, joined by poly‐*α*‐1,4‐D‐glucopyranoside bonds. The second component is amylopectin, which is a branched macromolecule in the polymeric chain, joined by linear poly‐*α*‐1,4‐D‐glucopyranoside and branched *α*‐1,6‐D‐glucopyranoside bonds [3]. Applications of native starches for food packaging are limited because of drawbacks such as their difficulty to water solubility and their rapid rate of retrogradation. To improve the quality of a given starch for selected applications, modifications to its polymeric chemistry could be conducted to increase its glass transmission temperature and thus decrease its tendency to retrograde at a given storage temperature. Crosslinking is a chemical modification method meant to stabilize starch granules, restrict overswelling and enhance its resistance to rupture caused by shearing and fluctuations in pH. Modified starch can be made to be more resistant to recrystallization, and this helps in maintaining its physical structure. Starch esterification is one of the crosslinking methods, which disrupts hydrogen bonding between its polymeric chains by replacing the hydroxyl groups with ester linkages [4]. This helps to reduce its hydrophilicity and thus its ability to form crystals and resultant retrogradation [5]. As a result, this paper investigated studies aimed at increasing the mechanical properties of a native starch. The intent was to also gain a better understanding of the factors involved in the development of starch‐based films and how they could be optimized.

pH is an important factor that affects the physiochemical properties of starches. Starch could be hydrolyzed at acidic conditions, and the product of acid modified starch is known as “acid converted starch” or “acid thinned starch.” Acidity as an approach to modify the properties of starch is an attempt to increase its flexibility, and this is best attempted at temperatures between 40°C and 60°C [6]. This is below its gelatinization temperature. During this process, the hydroxonium ions in the acidic solution will attack the oxygen atoms on the glycosides of the starch molecular chains and the glucosidic linkages will be broken. During this process, the acid will remain active but can be neutralized during posttreatment washing.

The literature reports that various testing techniques can be used to study the effects of pH on the properties of starches. Examples of these include x‐ray diffraction (XRD) as reported by Wang and Wang [7] and Fama´ et al. [8]; Fourier transformation infrared spectroscopy (FTIR) as reported by Lin et al. [9] and Reddy and Yang [10]; and thermal gravimetric analysis (TGA) reported by Jiugao et al. [11].

The mechanical properties of the edible films could be investigated by performing a tensile profile analysis (TPA) on material samples exposed to selected treatments. Chandak et al. studied the relationship between pH and the tensile properties of cross‐linked lotus seed starch films and found that sodium trimetaphosphate cross‐linked films showed a higher tensile strength (TS) and elastic modulus (EM) at pH 5.5 [12]. At the same time, Arrieta et al. [13]; Cao et al. [14] showed that cassava starch films had the highest mechanical yield resistance at pH 7.5. These studies showed that pH has an influence on the mechanical properties of starch films, but this varies depending on the source of the starch.

The main objective of this research was to investigate the influence of pH and how it affected the physiochemical properties of citric acid cross‐linked tapioca starch films. Figure 1 shows the experimental design of the research activities.

**Figure 1:**
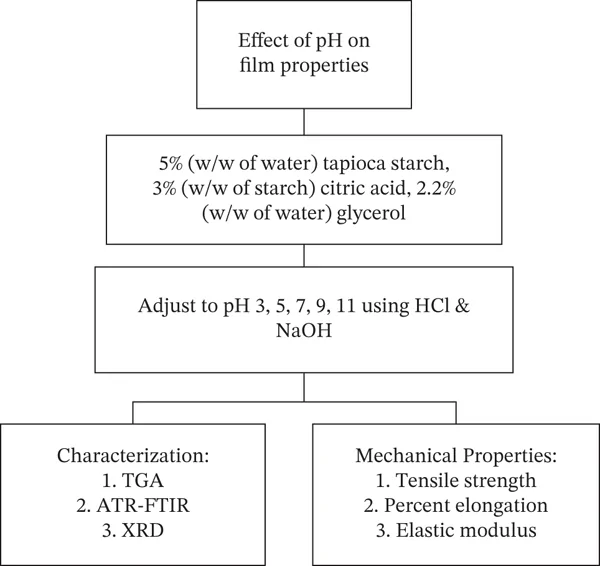
Experiment design.

## 2. Methods and Materials

### 2.1. Materials

These were pure (100%) gluten free modified tapioca starch (Gluten Free You and Me LLC. Columbus, Ohio). Citric acid (99.6%) obtained from Acros Organics (Fair Lawn, New Jersey), glycerol (Sigma‐Aldrich Co. St Louis, Missouri), hydrochloric acid (Fisher Chemical), 6.0 M sodium chloride (The Ohio State University Lab Stores‐Reagent, Columbus, Ohio).

### 2.2. Edible Film Formation

In this experiment, a process modified from Xu et al. was used for formation of the cross‐linked starch films [15]. The film forming solutions were prepared by dissolving 5% tapioca (*w*/*w* of water) and 3% (*w*/*w* of tapioca) citric acid in 80 g distilled water, and 2.2% glycerol (*w*/*w* of water) was added as a plasticizer. The film forming solutions were then adjusted to pH 3, 5, 7, 9 and 11 by sodium hydroxide and hydrochloric acid. Before heating, the starch solutions were stored at 23°C for 5 h to initiate the crosslinking reaction. To initiate gelatinization, the starch solutions were heated at 70°C–80°C for 15 min with stirring at 800 rpm.

Before casting, the starch solutions were ultrasonicated to remove bubbles. Aliquots of 60 g of the starch solutions were then poured into Teflon molds measuring 15.43 mm in diameter. These were then heated in an oven at 45^°^C ± 2^°^C for 12 h to produce dried films measuring approximately 0.1 mm in thickness. The thickness measurements were taken by a 280S‐10 Agd dial indicator Mitutoyo gauge (Aurora, Illinois). All films were conditioned at 23^°^C ± 1^°^C and 65% relative humidity (RH) for more than 1 day before being tested.

### 2.3. Attenuated Total Reflection Fourier Transformation Infrared Spectroscopy (ATR‐FTIR)

An ATR‐FTIR method was used to investigate the functional groups in the samples, and these were correlated with the degree of cross‐linking in the starch films. A Cary 630 FTIR (Agilent Technologies) with a diamond reflection crystal was used to conduct the testing. The scanning parameters were 5 Hz, 4 resolution, 32 background scans, and 64 sample scans. Each spectrum was collected in the mid‐infrared range at wavenumbers ranging from 650 to 4000 cm^−1^. The data for each sample were collected from three points.

### 2.4. XRD

The XRD measurement was conducted by using a Rigaku Miniflex600 x‐ray diffractometer to determine the morphology of the films and to estimate the degree of crystallinity. This allowed us to speculate about the retrogradation behavior of the films. The method used was a modification of one reported by Moreira et al. [16]. Slices from the film samples were attached to the sample holder in order to perform the test. The voltage of the x‐ray diffractometer was 40 kV and the current was set at 15 mA. The scanning angle was 3–30^0^ (2*θ*), and the sample holder was set at the speed of 1^0^/min. The scanning frequency was 0.01/1^0^.

### 2.5. TGA

The thermogravimetric analysis was conducted using a TGA Q50 thermogravimetric analyzer (TA Instrument Crop.) to determine the thermal stability of the films at the various pH levels. The method used was one reported by Lin and Pascall [17]. The graphs were generated by the Universal Analysis software package v4.5ATH. All specimens weighed 10 ± 0.5 mg and were placed in platinum pans. Each sample pan was flushed with 50 mm/Hg of pure nitrogen and heated from 25°C to 600°C at a rate of 15°C/min. The region between 25°C and 100°C was considered as water evaporation.

### 2.6. TPA

The mechanical properties of the starch film samples were analyzed according to the ASTM D882‐18 Standard Test Method for Tensile Properties of Plastics [18]. The testing was done using a Labthink Model XLW (EC) Universal Tester (Jinan, P.R. China). Prior to the analysis, the samples were cut into 100 × 25.4 mm strips then mounted onto the equipment. The grip distance was 50 mm, and the upper jaw was set to move at a crosshead speed of 50 mm/min. The data were collected by measuring three replicates and four testing of each replicate. All samples were conditioned at 23°C and 55% RH for 48 h before testing. All samples were analyzed for TS (MPa), percent elongation (%), and EM (Pa).

### 2.7. Statistical Analysis Methods

This experiment studied the influence of pH on the mechanical properties of cross‐linked starch films. Three parameters: TS (MPa), percent elongation (%), and EM (MPa) were tested to characterize the films. A one‐way factorial analysis of variance (ANOVA) was applied to evaluate the significance of five pH values (3, 5, 7, 9, and 11) on the starch films′ mechanical properties within the confidence interval at *p* < 0.05. The ANOVA was done using an SPSS program (IBM, Inc.).

## 3. Results and Discussion

### 3.1. ATR‐FTIR

Figure 2 shows the mid‐infrared FTIR spectra of the cross‐linked starch films formed at pHs of 3, 5, 7, 9, and 11. The spectra intensities for the ester linkages (‐COOR) in the cross‐linked starch films are located at a frequency around 1520 cm^−1^. This is supported in reports published by Ch′ng et al. [19]. The spectrum for the sample film at pH 3 has the lowest peak intensity for the ester linkage. One probable reason for this is that citric acid partially dissociates in aqueous solutions and thus it releases a limited amount of hydronium ions in such environments. The pKa value (25°C aqueous solution) of the first, second, and third steps of citric acid dissociation is 3.13, 4.67, and 6.40, respectively, as reported by Ksiazek [20].

**Figure 2:**
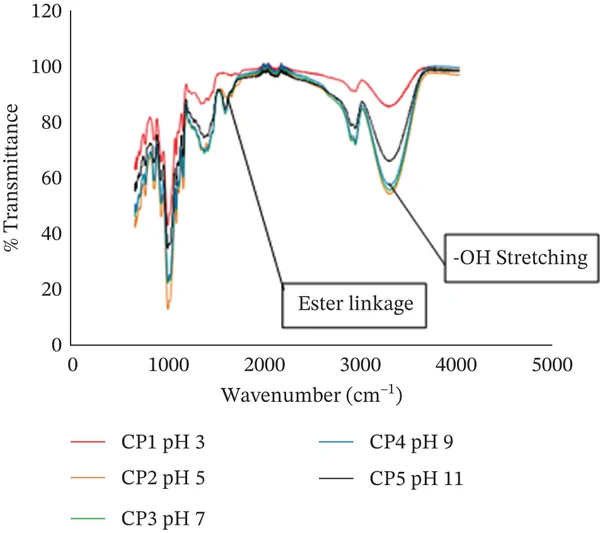
FTIR spectra of the cross‐linked starch films with pHs of 3, 5, 7, 9, and 11.

In our research, it appears that the degree of citric acid dissociation was lower than the dissociation equilibrium when the pH of the film‐forming solution was 3 and 5. Thus, there were limited quantities of citrate ions in the solution to react and bond with the hydroxyl groups on the starch polymeric chains. The signal intensity of the ester group (‐COOR) was the highest when the pH of the film forming solution measured 7. When the pH value exceeded 7, the degree of cross‐linking gradually decreased. The reason was possibly due to the number of hydroxides introduced by the NaOH. These may have been too excessive to be neutralized by the hydronium ions delivered by the citric acid. Thus, the hydronium ions were tied‐up by the alkaline hydroxide and were unavailable to bond with the hydroxyl groups on the starch molecule, and this resulted in the reduction in the degree of esterification. Ch′ng et al. reported that peaks located at ~3300 cm^−1^ are associated with –OH stretching [19]. The CP1 sample in our study, which had nearly no esterification, had the lowest signal intensity for the –OH group. According to Joshi et al., –OH stretching could be contributed by the free water content in starch films [21]. They also stated that the moisture content of cross‐linked starch that had been dehydrated at 50°C for 24 h in a sealed container was below 10%.

### 3.2. XRD

Figure 3 shows the XRD patterns of the cross‐linked starch films formed at pHs of 3, 5, 7, 9, and 11. The XRD patterns of these films were compared with those of a native starch film. The crystallinities of the citric acid cross‐linked starch varied at the different pH values of the film‐forming solutions. The native starch film showed the most intense peak height and the widest range of diffraction angle (2*θ*), indicating it had the highest crystallinity and the largest variability in crystal sizes. For the cross‐linked starch films, the crystallinities increased as the pH levels increased. The CP1 film, which was formed at pH 3 appeared to be the most amorphous and it had the lowest crystalline intensity and peak width. According to Wang and Wang, the crystallinities of corn, rice, and potato starches that had been acid thinned at pH 5.5 remained similar to that of native starch, although the crystallinity was slightly higher because of the acid treatment [7]. It should be noted that acids attack the granules of various starches differently. This is because of the compositional and structural differences in the chemistry of starches. For example, potato starch has mainly A‐type crystals, whereas for cassava starch, it is B‐type [22]. Additionally, the amorphous amylopectin regions can be more easily attacked by hydronium ions of the acid in solution. This could cause cleavage of the branches in the amylopectin and hence they could become more linear. However, fractured amylopectin is better able to reorder themselves and more easily convert into crystals at higher pH levels [7]. In addition, at lower pH levels, smaller starch molecules provide smaller crystals that would be harder to recrystallize. Since the starch in our study was cross‐linked by citric acid, it also helped to reduce the crystallinity due to the formation of hydrogels. This was so because crosslinking helps to increase network formation, swelling of the starch, induce moisture uptake, and thus hydrogel development. However, crystallinity tends to reduce polymeric swelling, and this does not promote hydrogel formation. The three‐dimensional structure of the hydrogels provided the starch with a greater ability to retain moisture and this influenced the starch towards an amorphous nature [23]. When the pH of the film forming solutions increased to pH 11, the increasing concentrations of the hydroxides more readily provided the starch molecules with better protection from being hydrolyzed by the reaction of the hydronium ions. This thus influenced the formation of a higher degree of crystallinity in the films. Similar results were obtained by Sakkara et al., when they investigated the effect of pH on the physicochemical properties of starch films [24].

**Figure 3:**
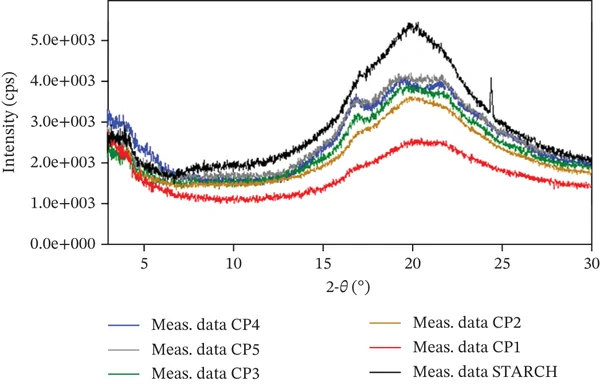
XRD patterns of cross‐linked starch films: CP1‐pH 3, CP2‐pH 5, CP3‐pH 7, CP4‐pH 9, and CP5‐pH 11.

### 3.3. TGA

The TGA of the samples was conducted from 25°C to 600°C to evaluate the influence of pH on the thermal stability of the citric acid crosslinked starch films. The TGA results are shown in Figure 4, and they are heat‐related weight changes in the films as a function of temperature. The derivatives of these changes are shown in Figure 5. According to the rate of weight loss, the weight percentage/temperature curves could be divided into three sections: (1) 25°C–260°C; (2) 260°C–350°C; and (3) 350°C–600°C.

**Figure 4:**
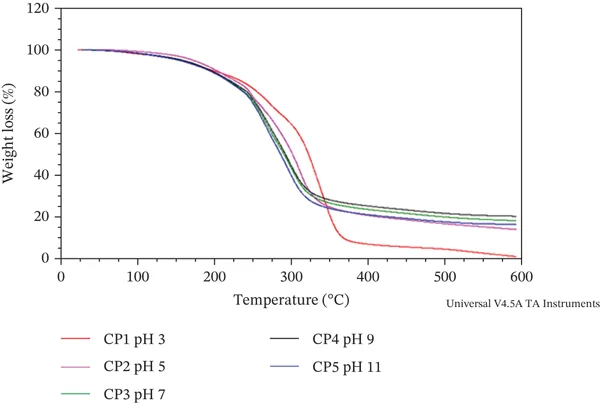
The influence of pH on the heat related weight loss of cross‐linked starch films.

**Figure 5:**
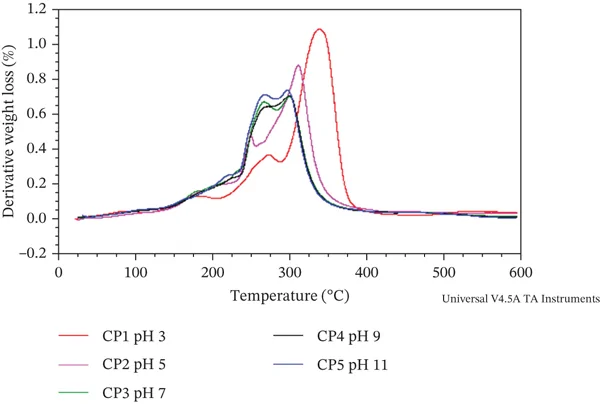
Starch film weight loss versus temperature derivative curve.

The results in Figure 5 show that two substantial peaks were present in the temperature region between 200°C and 400°C. In this region, the weight losses were the greatest for all samples. The first peak region located between 200°C and 275°C related to the evaporation of volatiles made up of glycerol and a part of the structural water in the film samples [25]. The second peak region located between 275°C and 400°C related to the physical decomposition of starch as reported by Reddy and Yang [10]. The separation of the two peaks became more significant as the pH of the film formation solution decreased. The CP1 sample, which was formed at pH 3, had the most apparent separation of the two peaks. As the pH values of the film formation solutions became higher, the first and second temperature regions became increasingly merged. The temperature region for the CP4 sample had the highest degree of overlap. According to the FTIR results, the cross‐linked films formed at neutral and alkaline conditions had higher degrees of cross‐linking as shown by their higher ester linkages. This result may have occurred because these films probably had a stronger hydrogel structure due to their alkaline condition, and this thus induced a higher degree of cross‐linking when compared with the films made at a lower pH. Xiao reported that hydrogels can help certain starches improve their ability to retain fluids, including moisture [23]. In our experiment, it is thus plausible to conclude that the combination of moisture, glycerol, and cross‐linking contributed to the overlap of the weight change temperature regions. The location of the starch physical decomposition region shifted to a higher temperature when the films were formed in a more acidic condition. It was found in this experiment that the starch decomposition temperature negatively correlated with the increasing degree of cross‐linking. Menzel et al. reported that cross‐linking helped to improve the molecule weight of starches [26]. As a result, increasing the molecule weight of cross‐linked starches would reduce its weight loss percentage when compared with starches having lower molecular weights.

Figure 6 shows the weight changes in the starch films from 25°C to 260°C. Except for sample CP2, all other films began losing weight at around the initial 25°C temperature. The remaining weight of the residue from the CP1 film at 260°C was the highest, and it gradually became lower when the pH of the films became higher. This indicated that the thermal resistance of the cross‐linked films appeared reduced when the alkalinity of the film‐forming solution became stronger during this region.

**Figure 6:**
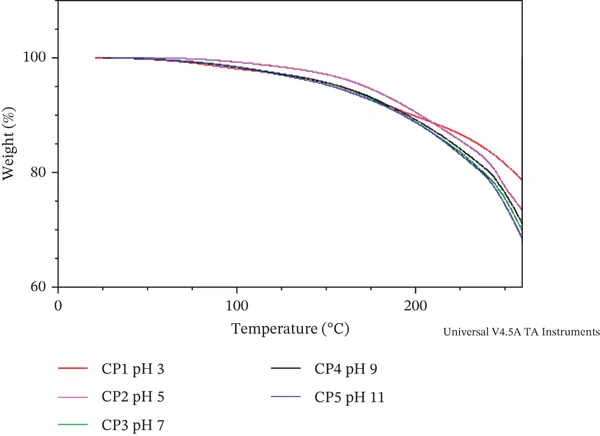
Heat‐related weight change between 25°C and 260°C.

Contrary to what was shown in the results from the 25°C to 260°C temperature range, a reversal of this trend was seen in the results for increasing pH levels during the 260°C–350°C range (Figure 7). As was mentioned earlier, this increasing pH had a positive influence on the thermal stability of the crosslinked starch film. This 260°C–350°C temperature range is where starch decomposition occurs. The rate of weight loss in the acidic films exceeded those of the neutral and alkaline films at about 320°C. A similar result was found by Reddy and Yang who reported a higher thermal stability in starch films cross‐linked with citric acid when compared with nonmodified films at around 320°C [10]. It was assumed that the increase in the molecular weight of cross‐linked starch films reduced the resistance to heat‐related changes below the temperature at approximately 320°C, whereas it improved the thermal stability when the temperature increased above 320°C. Wang and Wang observed a reduction in the glass transition temperature and enthalpy of acid‐thinned starch when compared with the native starches. The acid hydrolysis mainly took place in the amorphous amylopectin region of the starches and slightly increased the crystallinity of the acid‐thinned samples [7]. This increased crystallinity could be a possible reason for the comparatively higher thermal resistance of the acidic starch films when compared with the alkaline films within the temperature region below 320°C.

**Figure 7:**
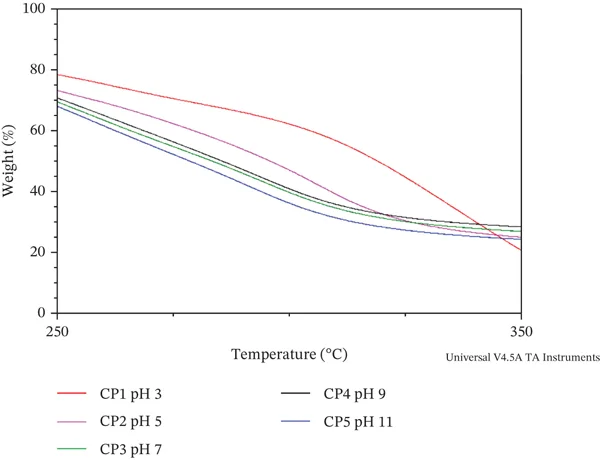
Heat‐related weight change between 260°C and 350°C.

This behavior (at < 320°C) can be explained by the fact that acid treatment tends to increase crosslinking and limits mobility of the polymeric chains. This limits crystals formation and the ability of the starch to tolerate higher temperatures. Also, the acid treatment increases the formation of ester linkages within the starch. When this occurs at temperatures < 320°C, the ester linkages tend to be disrupted before that of the glucosidic backbone of the starch, and it is displayed as a weight loss in the TGA curves. However, at temperatures above 320°C, the reduction in weight loss occurs because of the cross‐linking and covalent bonding between the polymeric chains, and this serves to increase the energy required to degrade the starch. This slows degradation of the starch and its weight loss. Thus, in a TGA analysis, the cross‐linked film may begin degrading below < 320°C, but it ultimately retains more mass at elevated temperatures above 320°C [27–29].

Figure 8 shows the weight changes of the cross‐linked starch films from 350°C to 600°C. The heat‐related changes in this region are related to the starch′s chemical decomposition (carbonization) [30]. The trend of the pH influence on the thermal resistance of the cross‐linked starch films remained similar to what was shown in Figure 7. The starch film formed at pH 3 showed the most significant weight loss as the residual weight was below 5% of the initial weight. The sample formed at pH 9 appeared to have the optimized heat resistance up to 600°C. The strong alkaline condition (pH 11) reduced the thermal stability of the films. This was possibly due to the influence of the excessive hydroxides from the strong alkaline (NaOH) and its increased activity in the starch molecules. This may have decreased the amount of energy required to decompose these starch films.

**Figure 8:**
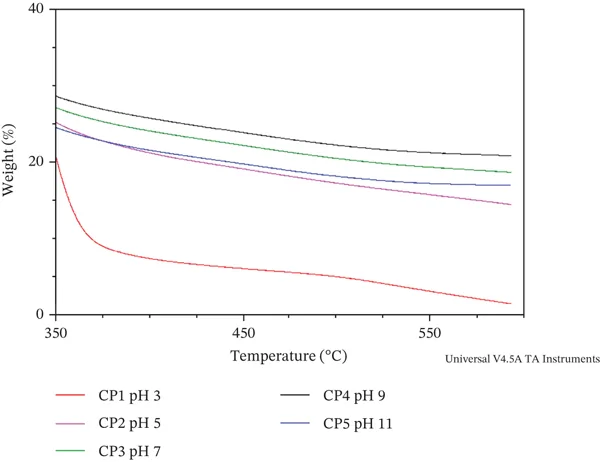
Heat‐related weight change between 350°C and 600°C.

### 3.4. TPA

Figure 9 shows the TS and percent elongation for the starch films processed at the varying pH conditions. The results show that the TS of the cross‐linked starch film formed at pH 3 had the lowest value at 2.997 MPa, whereas pH 9 provided the optimized TS at 20.022 MPa. This reduction in TS as the pH level decreased probably occurred because of cleavage to the polymeric chains in the higher acidic environment. Menzel et al. reported that such cleavage would result in a lowering of the molecule weight of the polymer and concurrently a lowering of the TS of such films [26]. Menzel et al. confirmed this when they used high‐performance size‐exclusion chromatography to measure the molecular weight of starch films made at varying degrees of acidity [26]. This showed that small molecules with less crosslinking would be more flexible and at the same time be more elastic and show a greater degree of elongation to an external tensile force [31].

**Figure 9:**
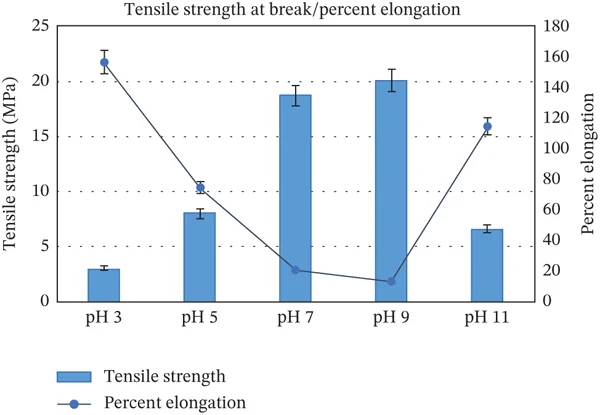
Effect of pH value on tensile strength (MPa) and elongation (%) of cross‐linked starch films.

Thus, it could be concluded that the acidic conditions in our study gave rise to cross‐linked films with low TS and lower molecule weight. The FTIR results confirmed that the films formed at pH 3 and 5 had lower degrees of esterification and concurrently lower degrees of cross‐linking and TS. Additionally, the ANOVA showed that increasing the pH from 3 to 7 significantly (*p* < 0.05) increased the TS of the starch films. The statistical results also showed that there was no significant difference (*p* > 0.05) in TS at pH 7 and 9. For the film formed at pH 11, there was a significant (*p* < 0.05) decrease in TS when compared with the results of the films at pH 7 and 9. These results led us to conclude that pH 7 or 9 could be optimum for maximizing the TS of the film samples.

For the percent elongation, the results are an inverse mirror of the results for the TS. This is so because an increase in cross‐linking decreases segmental mobility of the polymeric chains and thus the ability to flex is reduced [32]. This causes the materials to become less elastic. However, Figure 9 shows that at pH 11, a significant (*p* < 0.05) increase in elongation occurred at the higher alkalinity of the film forming solution.

The bar chart in Figure 10 shows the effect of pH on the EM of the cross‐linked starch films. The EM showed a positive correlation with TS. This shows that the EM is similarly affected by the pH of the film forming solution. EM is a measure of the stiffness of the films. It provides information on how much the films will resist deformation when exposed to an applied stress. This property of a material is reversible once the applied stress is removed. It can be obtained from the linear part of the slope of a stress‐strain curve [33]. The results showed that the EM of the films was optimized at pH 9, which was 20 times that of the EM of the film formed at pH 3.

**Figure 10:**
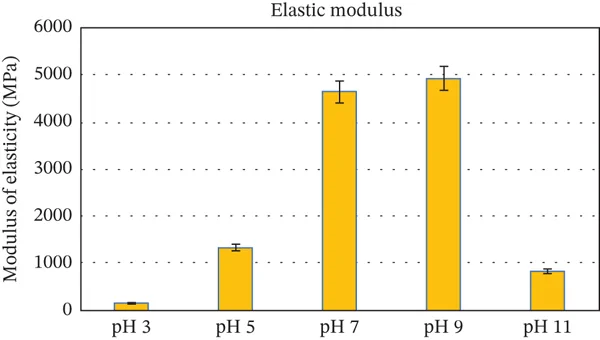
Effect of pH concentration on elastic modulus of cross‐linked starch films.

## 4. Conclusion

From the results obtained in this study, it could be concluded that pH had a significant impact on the intensity of ester linkages in cross‐linked starch films. The ATR‐FTIR results showed that the intensity of these ester linkages in cross‐linked starches formed at acid conditions were lower than that of the starches formed at neutral and alkaline conditions. From the XRD patterns, it was concluded that increased acidity of the film‐forming solution produced films with lower crystallinity. The TGA results showed that pH 9 provided the cross‐linked starch film with optimized heat resistance. The TPA result showed that pH directly influenced the TS, percent elongation, and EM of the starch films. The TS of the starch film formed at pH 9 was 20 times higher than that of the film formed at pH 3. However, strong alkaline conditions (at pH 11) reduced the thermal stability and TS of the crosslinked starch films.

## Author Contributions

Yuan Chi: data collection, formal analysis, and original draft. Melvin A. Pascall: conceptualization, investigation, methodology, validation, supervision, and funding acquisition.

## Funding

No funding was received for this manuscript.

## Ethics Statement

Ethics approval was not required for this research.

## Conflicts of Interest

The authors declare no conflicts of interest.

## Data Availability

The data that support the findings of this study are available from the corresponding author upon reasonable request.
